# Oculomotor compressive neuropathy secondary to calcifying pseudoneoplasm of the neuraxis (CAPNON)

**DOI:** 10.1093/jscr/rjae507

**Published:** 2024-10-11

**Authors:** Verónica Alzate-Carvajal, Humberto Jose Madriñán-Navia, Luis Alberto Escobar, Camilo E Moreno-Huertas

**Affiliations:** Neurocirugía, Facultad de Ciencias de la Salud, Universidad Icesi, Cali 760008, Colombia; Neurocirugía, Fundación Valle del Lili, Cali 760026, Colombia; Departamento de Patología, Fundación Valle del Lili, Cali 760026, Colombia; Facultad de Ciencias de la Salud, Universidad Icesi, Cali 760008, Colombia; Neurocirugía, Fundación Valle del Lili, Cali 760026, Colombia

**Keywords:** calcifying pseudoneoplasm of the neuraxis, cranial nerve palsy, skull base tumor

## Abstract

Calcifying pseudoneoplasm of the neuraxis (CAPNON) is an uncommon entity and a rare cause of third cranial nerve palsy. We review the case of a 17-year-old male with a 9-month history of progressive left third cranial nerve palsy. Cerebral magnetic resonance image showed a left clinoidal lesion with low signal intensity in T2 and T1 sequences with signs of calcification in the computed tomography and without vascular lesion in AngioMRI. A left pterional approach was performed with posterior clinoidectomy and total resection of the lesion. Calcifying pseudoneoplasm of the neuraxis is an infrequent pathology that presents in the skull base and spine that requires surgical treatment in the presence of compressive phenomena and differential diagnosis as meningioma, chordoma, and metastasis should be considered.

## Introduction

Calcifying pseudoneoplasm of the neuraxis (CAPNON) is a rare, fibrous, non-neoplastic lesion with an unclear etiology [1]. The symptoms vary depending on the location and compressive effect on structures in the spine, skull base, or posterior fossa, and include seizures, headache, motor or sensory disturbances, and cranial nerve palsy [1, 2].

The first case, reported in 1978, was described as atypical bone metaplasia [2]. Since then, ~150 cases have been reported, and in certain instances, it has been linked to other inflammatory lesions or tumors [3].

The cornerstone of treatment is complete surgical resection to resolve the compressive effect [4]. The diagnosis relies on histopathological criteria.

We present the case of a young male patient with third cranial nerve palsy secondary to CAPNON.

## Case presentation

A 17-year-old healthy male presented to clinics with a 9-month history of progressive left ptosis, diplopia, and gaze disturbance. The neurological assessment evidenced complete left oculomotor palsy. Visual acuity and field examination were normal. The rest of the neurologic exam was unremarkable.

**Figure 1:**
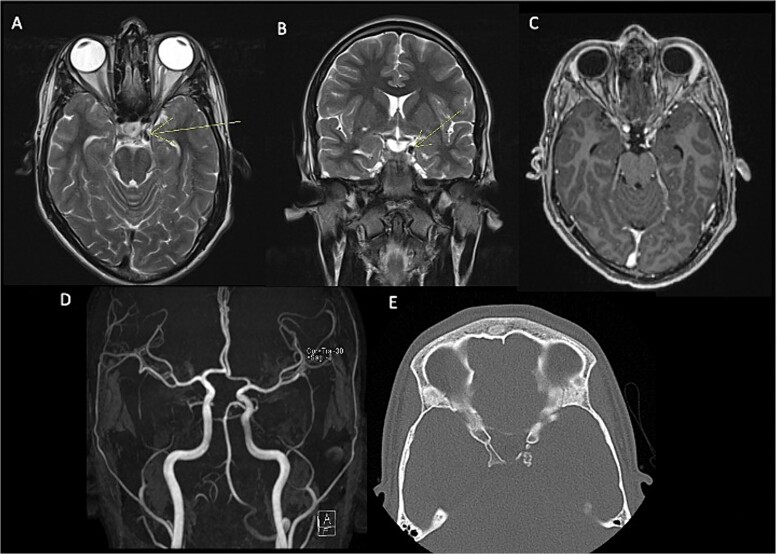
Pre-surgical MRI (A–C) showing the presence of a lesion with low signal intensity on T2-weighted sequences (A, B) of 0.7 × 0.6 cm adjacent to anterior clinoid process and tents, concerning the oculomotor left cistern, with peripheral enhancement in post-contrast sequences (C) contacting the left third cranial nerve. No evidence of aneurysms in angiogram MRI (D). Head CT scan (E) with a calcified exophytic lesion concerning the left anterior clinoid.

In the initial assessment of painless oculomotor palsy, brain MRI revealed a small, rounded lesion adjacent to the left posterior clinoid process (Fig. 1). Subsequent angiography (MRA) was performed due to suspicion of vascular pathology, confirming a well-defined lesion with low signal intensity in both T1 and T2 sequences. Post-contrast T1-weighted images demonstrated thin peripheral enhancement around the lesion. Aneurysms were ruled out by MRA, and a follow-up head CT scan identified a calcified lesion on the left posterior clinoid process extending into the interclinoid ligament, situated adjacent to the left oculomotor cistern. Additionally, diffuse calcification of the interclinoid ligaments was observed on CT imaging.

A left pterional craniotomy was performed, with intradural peeling of the middle fossa from the lateral edge of the superior orbital fissure, exposing the anterior clinoid process. Subsequently, a left transsylvian approach was undertaken, revealing the left oculomotor triangle. A small, calcified lesion adhered to the left posterior clinoid process, resulting in compression and infiltration of the third cranial nerve. Intradural resection of the nodular lesion of the posterior clinoid was performed, involving a segment of the third cranial nerve that was infiltrated by the lesion. Additional mobilization of the posterior choroidal artery was necessary, accompanied by coagulation of perforating vessels supplying the tumor.

**Figure 2:**
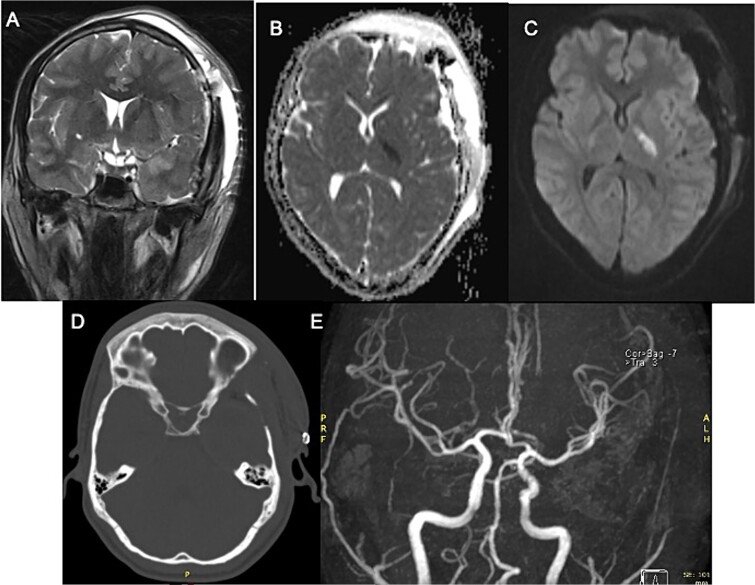
Postoperative MRI with left third cranial nerve decompression in T2-weighted sequence (A) and a high DWI signal and low ADC signal in the posterior limb of left internal capsule (B, C) with no occlusion in angiogram MRI demonstrating ischemic event (E) Head CT Scan (D) without compressive lesion.

Postoperatively, the patient developed progressive right-sided hemiparesis and altered consciousness progressing to stupor, attributed to vasospasm of the left anterior choroidal artery. Evidence of arterial narrowing was observed in the postoperative angiography with MR (Fig. 2E). No endovascular chemical angioplasty was performed due to ischemic findings on FLAIR sequence. Following rehabilitation, the patient was discharged home with improved strength ($ \frac{4}{5} $).

**Figure 3:**
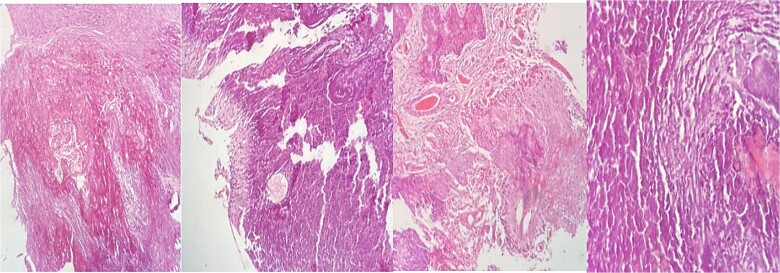
Hematoxylin–eosin (HE) staining with extensive calcified tissue is recognized. There are areas of myxoid tissue, focal piloid proliferation, arachnoid cellularity, and mononuclear inflammatory cells.

Pathology (Fig. 3) reported a calcified tissue with spindle cell proliferation without neoplasic features, and complementary immunohistochemistry of the calcified material was positive for enolase and D240 and negative for inhibin and Olig 2. CD34 was limited to some vessels.

## Discussion

The third cranial nerve palsy due to a compressive effect is a frequent finding in neurosurgical practice, most of the time caused by tumors, aneurysms, or traumatic brain injury. However, it is important to denote other infrequent etiologies like CAPNON, which is a reactive lesion that generates the proliferation of spindle cells and myxoid matrix with an associated calcification, generally adjacent to dura mater or arachnoid [5, 6]. These lesions can also be present in the posterior fossa or the spine [7].

Approximately 18.6% of CAPNON cases are incidental findings discovered during autopsies [8]. Seizures are the most prevalent symptoms, occurring in 31.6% of cases [3]. When the location is at the skull base, the most common finding is cranial neuropathy, with the trigeminal, hypoglossal, and accessory nerves being most frequently affected [5].

The pathogenesis of CAPNON involves various hypotheses, including reactive processes like metaplastic transformation, neoplasms, and degenerative changes [5]. The prevailing theory suggests a proliferative response linked to inflammation, often associated with diverse underlying conditions, such as vascular and neoplastic factors. Evidence supporting a preference for axonal tissue includes the presence of neurofilament light chain protein (NF-L) within the tumor, which may explain axonal involvement and neuroaxis calcification [5, 6].

During surgical exploration in our case, we observed an infiltrative lesion adjacent to the third cranial nerve characterized by inflammatory and neovascular tissue. MRI typically reveals a well-defined, rounded lesion with low intensity on T1 and T2 sequences and uniform enhancement on post-contrast images. CT scans show calcification involving bone or meningeal surfaces [3, 7, 8].

Differential diagnosis depends on whether the lesion is intra- or extradural. Intra-dural possibilities include meningioma, chordoma, chondrosarcoma, metastasis, and schwannoma, while extradural considerations often include calcified metastasis and cavernous malformations [9, 10].

In contrast, vasospasm following neurosurgical procedures is a rare complication typically associated with subarachnoid hemorrhage. Proposed mechanisms include irritative effects from lytic and inflammatory blood products similar to ruptured aneurysms, along with direct mechanical irritation of vascular smooth muscle cells and vasa nervorum, especially in skull base tumors [11].

Chemical endovascular angioplasty has shown promising results in improving arterial caliber and neurological deficits in reported cases following procedures, such as transsphenoidal pituitary tumor resection [12] and sphenoclinoidocavernous meningioma [13]. However, in our case, due to noted ischemic changes at diagnosis, we opted against this intervention, and the patient demonstrated significant improvement in strength during follow-up.

In conclusion, third cranial nerve palsy can arise from various compressive causes, including rare entities like CAPNON. While diagnostic imaging plays a crucial role in identifying these lesions, it remains challenging to differentiate them definitively based solely on imaging findings.
